# Supplementary material for: A Coil-to-Helix Transition Serves as a Binding Motif for hSNF5 and BAF155 Interaction
Source: Int J Mol Sci. 2020 Apr 1;21(7):2452. doi: 10.3390/ijms21072452 (PMC7177284; doi:10.3390/ijms21072452)
Supplement: Supplementary file 1 [file ijms-21-02452-s001.pdf]

International Journal of *Molecular Sciences*

MDPI

Supplementary

A Coil-to-Helix Transition Serves as a Binding Motif for hSNF5 and BAF155 Interaction

Jeongmin Han [1,†], Iktae Kim [2,†], Jae-Hyun Park [1], Ji-Hye Yun [1], Keehyoung Joo [3], Taehee Kim [1], Gye-Young Park [1], Kyoung-Seok Ryu [4], Yoon-Joo Ko [5], Kenji Mizutani [6], Sam-Young Park [6], Rho-Hyun Seong [7], Jooyoung Lee [8,*], Jeong-Yong Suh [2,*] and Weontae Lee [1,*]

1 Structural Biochemistry & Molecular Biophysics Laboratory, Department of Biochemistry, College of Life Science and Biotechnology, Yonsei University, Seoul, 120-740, Korea.; jmhan723@yonsei.ac.kr (J.H); jhpark@spin.yonsei.ac.kr (J.-H.P.); jihye2@spin.yonsei.ac.kr (J.-H.Y.); thkim@spin.yonsei.ac.kr (T.K); gypark@spin.yonsei.ac.kr (G.-Y.P.);
2 Department of Agricultural Biotechnology and Research Institute of Agriculture and Life Sciences, Seoul National University, 1 Gwanak-ro, Gwanak-gu, Seoul, 08826, Korea.; iktaekim@snu.ac.kr (I.K)
3 Center for In Silico Protein Science and Center for Advanced Computation, Korea Institute for Advanced Study, Seoul, 130-722, Korea.; newton@kias.re.kr (K.J.)
4 Division of Magnetic Resonance Research, Korea Basic Science Institute, Yangcheong-Ri 804-1, Ochang-Eup, Cheongwon-Gun, Chungcheongbuk-Do, 363-883, Korea.; ksryu@kbsi.re.kr (K.-S.R.)
5 National Center for Inter-University Research Facilities, Seoul National University, 1 Gwanak-ro, Gwanak-gu, Seoul, 08826, Korea.; yjko@snu.ac.kr (Y.-J.K)
6 Drug Design Laboratory, Graduate School of Medical Life Science, Yokohama City University, Tsurumi, Yokohama, Japan.; mizutani@yokohama-cu.ac.jp (M.K.); park@yokohama-cu.ac.jp (S.-Y.P)
7 Department of Biological Sciences, Institute of Molecular Biology and Genetics, Research Center for Functional Cellulomics, Seoul National University, Seoul, 151-742, Korea.; rhseong@snu.ac.kr (R.-H.S.)
8 Center for In Silico Protein Science and School of Computational Sciences, Korea Institute for Advanced Study, Seoul, 130-722, Korea.
* Correspondence: jlee@kias.re.kr, jysuh@snu.ac.kr, and wlee@spin.yonsei.ac.kr
† These authors contributed equally.

Supplementary Materials

Figure S1. The electrostatic surface charged models of hSNF5$^{171-258}$ and BAF155^{SWIRM}

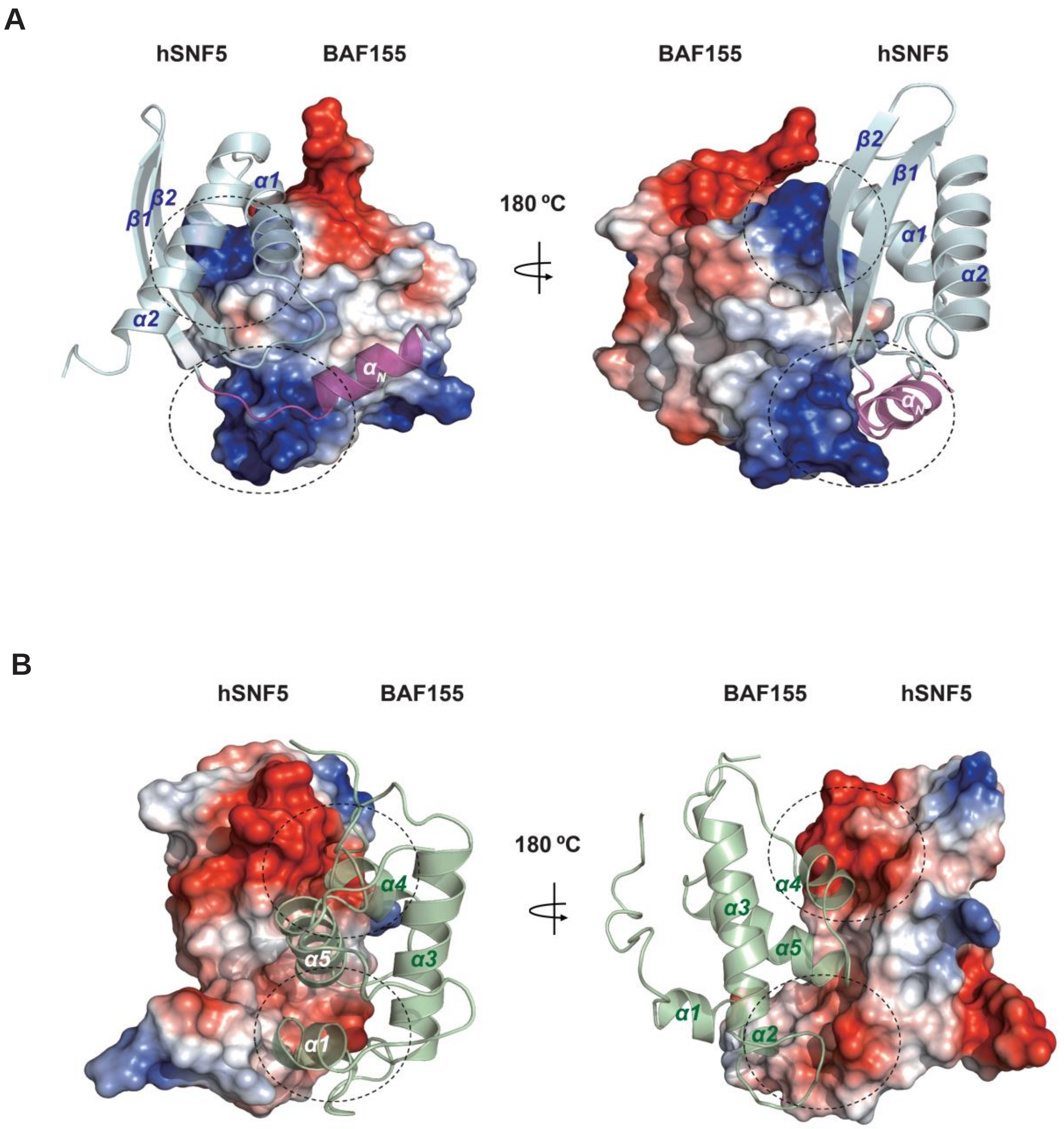

Figure 1. The electrostatic surface charged models of hSNF5$^{171\text{-}258}$ and BAF155^{SWIRM}. (**A**) Surface charge models represent that hSNF5$^{171\text{-}258}$(cyan ribbon model) binding site on BAF155^{SWIRM} has positive charges. (**B**) The surface of hSNF5$^{171\text{-}258}$ complexed with BAF155^{SWIRM}(green ribbon model) has negative charges. The electrostatic potential surfaces were calculated using the APBS program**.**
